# The Preparation of Nano-SiO_2_/Dialdehyde Cellulose Hybrid Materials as a Novel Cross-Linking Agent for Collagen Solutions

**DOI:** 10.3390/polym10050550

**Published:** 2018-05-21

**Authors:** Cuicui Ding, Yang Zhang, Binhan Yuan, Xiaodong Yang, Ronghui Shi, Min Zhang

**Affiliations:** 1 College of Ecological Environment and Urban Construction, Fujian University of Technology, Fuzhou 350108, China; dingcuicui.1124@163.com (C.D.); 18850495318@163.com (Y.Z.); 15123750803@163.com (B.Y.); 18250162677@163.com (X.Y.); shironghui@fjut.edu.cn (R.S.); 2 College of Materials Engineering, Fujian Agriculture and Forestry University, Fuzhou 350002, China

**Keywords:** dialdehyde cellulose, nano-silicon dioxide (nano-SiO_2_), collagen, hybridization, flowability, thermal stability

## Abstract

Nano-SiO_2_ was immobilized onto dialdehyde cellulose (DAC) to prepare SiO_2_/DAC hybrid materials. Fourier transform infrared spectra (FTIR), thermogravimetric analysis and field emission scanning electron microscopy of SiO_2_/DAC indicated that nano-SiO_2_ had been successfully hybridized with DAC. X-ray diffraction suggested that the structure of DAC was influenced by the nano-SiO_2_. SiO_2_/DAC was then used as the cross-linker of collagen solutions. Gel electrophoresis patterns and FTIR reflected that cross-linking occurred between DAC and collagen, but that collagen retained the native triple-helix, respectively. Differential scanning calorimetry indicated that the thermal stability of collagen could be effectively improved by SiO_2_/DAC. Dynamic rheology tests revealed that the flowability of collagens cross-linked by SiO_2_/DAC was superior to that of those cross-linked by DAC; meanwhile, collagens cross-linked by SiO_2_/DAC possessed a more homogeneous morphology compared to those cross-linked by DAC. The hybridization of SiO_2_/DAC as a cross-linker for collagen could effectively prevent the gelation caused by excessive cross-linking, and significantly improve the thermostability of collagen, which could be helpful for collagen being applied in fields including biomaterials, cosmetics, etc.

## 1. Introduction

Collagen, the major component of extracellular matrices, has become a popular biomaterial and has been widely used in pharmacy, food, cosmetics and tissue engineering due to its bioactivity, biocompatibility and biodegradability [1,2,3]. Usually, aqueous preparations of collagen can be used for medial injection [4] or used as drug carriers and so on [5,6]; additionally, collagen solutions may also be processed into a variety of formats, including sheets, fibers, sponges and packaging films [7,8,9,10]. During these applications and processes, the thermal stability of collagen in solutions plays an essential role in keeping collagen against denaturation, as the excellent characteristics of the native collagen would be lost if the collapse of the triple helix to a random coil occurred. 

The thermal stability of collagen in solutions has been studied widely, and all of these studies suggest that collagen has a relatively weak thermal stability, which limits its industrial and biological applications. Chemical cross-linking procedures are a useful method for reinforcing the collagen structure, and several cross-linkers, such as glutaraldehyde (GA) [11], formaldehyde [12] and diisocyanates [13], have been used. However, these cross-linkers usually show toxicity. Some researchers have developed cross-linking agents with low toxicity, such as carbodiimide [14], but nevertheless, incomplete cross-linking under a sufficiently high carbodiimide concentration could be caused by side reaction of the carbodiimide [15]. Therefore, it was necessary to develop an alternative cross-linking agent with good biocompatibility and low cytotoxicity.

Cellulose is the most abundant natural polymer and has some fascinating properties, such as biocompatibility, biodegradability and desirable mechanical properties, and thus a variety of cellulose derivatives that can be widely used in many fields have been produced by chemical modifications such as esterification, halogenation, oxidation and etherification [16], or by chemical grafting [17]. Amongst these derivatives, periodate oxidized cellulose is prepared by specific cleavage of the C2–C3 bond of the glucopyranoside ring, producing two aldehyde groups per unit [18]. Thus, it is named as dialdehyde cellulose (DAC), which is an open chain polymer containing aldehyde groups. Considering the functional aldehyde groups could produce cross-links with free amino groups, DAC can be used as a novel cross-linking reagent for collagen. Kanth et al. investigated the effect of DAC on cross-linking efficiency, hydrothermal and enzymatic stability of collagen fibers using DAC as a cross-linker. The results suggested that the hydrothermal stability of collagen fibers could be improved significantly [19]. However, as for collagen in solutions, a solid-like network structure would be gradually developed, and thus gelation is prone to occur when cross-linking reagent is added [20]. In particular, compared to cross-linkers with low molecular weight, the gelation of collagen solutions was more difficult to avoid, due to the presence of a large number of aldehyde groups in the long chain of DAC. Hence, DAC needs to be modified in order to maintain the flowability of the cross-linked collagen in solutions. 

SiO_2_, which was formed by the hydrolysis of tetraethoxy silane, has been introduced into leather to improve the hydrothermal stability [21]. With collagen as a model of leather, it was revealed that hydrogen bonding between the silanol of the silica and the carboxyl hydroxyl, as well as amino groups of collagen, occurred between SiO_2_ and collagen [22]. Obviously, this effect was weaker than covalent cross-linking induced by DAC, so the gelation of collagen solutions could be expected to be inhibited if part of the aldehyde group of DAC were replaced by SiO_2_; meanwhile, the thermal stability of collagen solutions could still be improved by the effects from both DAC and SiO_2_. Therefore, in the present work, we attempted to prepare SiO_2_/DAC hybrid materials via organic/inorganic hybridization of nano-SiO_2_ and DAC to be a novel cross-linking agent for collagen. 

In the present study, the structure and morphology of the SiO_2_/DAC hybrid materials were investigated by the methods of Fourier transform infrared (FTIR), X-ray diffraction (XRD) and thermogravimetric analysis (TGA). Then the hybrid materials were used as a novel cross-linking agent for collagen solutions. Subsequently, the modified collagens were characterized, and the effect of modification was evaluated via sodium dodecyl sulphate-polyacrylamide gel electrophoresis (SDS-PAGE), differential scanning calorimetry (DSC), oscillatory rheological measurements and Field emission scanning electron microscopy (FESEM). New insights into and understanding of the mechanism of stabilization of collagen by SiO_2_/DAC hybrid materials via organic/inorganic hybridization of nano-SiO_2_ and DAC were gained.

## 2. Materials and Methods 

### 2.1. Materials 

Collagen was self-prepared from calf skins according to our previous work [23]. Briefly, the supernatants extracted from the delimed and neutralized bovine split pieces by 0.5 mol/L acetic acid containing 3% pepsin (1:3000) were collected by refrigerated centrifugation at 9000× *g*. After this step, the supernatants were salted out by the addition of NaCl to a final concentration of 0.7 mol/L, and then the precipitate was again dissolved in 0.5 mol/L acetic acid and dialyzed against 0.1 mol/L acetic acid for 3 days. Finally, pepsin-soluble collagen solution (about 3 mg/mL) was lyophilized by a freeze dryer (Labconco Freeze Dryer FreeZone 6 Liter, LABCONCO Co., Kansas City, KS, USA) at −50 °C for 2 days and stored at 4 °C until used.

Microcrystalline cellulose (degree of polymerization = 200) was purchased from Aladdin Co., Ltd. (Shanghai, China). Sodium periodate (NaIO_4_), N-(β-aminoethyl)-γ-aminopropyl trimethoxy-silane (AEAPTS) and tetraethyl siloxane (TEOS) with analytical pure grade were purchased from National Pharmaceutical Group (Shanghai, China).

### 2.2. Preparation of SiO_2_/DAC Hybrid Materials

Dialdehyde cellulose (DAC) was prepared according to the methods reported earlier, with modifications as described [24]. Microcrystalline cellulose (25 g) was hydrolyzed in 5 mol/L hydrochloric acid for 12 h. The hydrolyzed cellulose was suspended in deionized water, and then 25 g of sodium periodate was added, stirring for 4 h with a magnetic stirrer. The pH of the solution was maintained at 3.5 during the reaction and the reaction was performed in the dark at 40 °C. Ethylene glycol (5 mL) was added to terminate the reaction, and then the product was extracted with centrifugation in t-butyl alcohol for three times. Finally, the resultant was lyophilized by the freeze dryer at −50 °C for 2 days. The obtained DAC were converted to nitrogen-containing compounds using the Schiff base reaction with hydroxylamine hydrochloride, according to the method of reported previously [25]. Elemental composition of nitrogen-containing derivatives was determined for C, H, and N by an elemental analyzer (Elementar Vario EL, Elementar Co., Hanau, Germany), and then the dialdehyde content was calculated to be 21.2%.

TEOS (50 mL) was added to a 50% (*v*/*v*) ethanol solution (250 mL); after stirring at room temperature for 1 h, glacial acetic acid (2.5 mL) was added and then stirred at 75 °C for 15 min. After this step, AEAPTS was dropwise added into the solution until a gel was formed. The gel was washed with 50% (*v*/*v*) ethanol solution for three times, and then SiO_2_–NH_2_ was lyophilized by the freeze dryer at −50 °C for 2 days.

The SiO_2_/DAC hybrid materials were then prepared as follows: a mixture was prepared by dispersed SiO_2_-NH_2_ and DAC together in 50% (*v*/*v*) ethanol solution with the mass ratios (SiO_2_/DAC) of 1/20, 1/10, 1/5 and 1/2, respectively. After stirring at 65 °C for 18 h, the resultant was washed by deionized water three times, and then hybrid materials were obtained by lyophilization with the freeze dryer at −50 °C for 2 days and were stored at 4 °C until use. The hybrid materials were denoted as SiO_2_/DAC (1/20), SiO_2_/DAC (1/10), SiO_2_/DAC (1/5) and SiO_2_/DAC (1/2), respectively, according to the initial mass ratios of SiO_2_/DAC. The nano-SiO_2_ in the following description was called SiO_2_ for short.

### 2.3. Characterization of SiO_2_/DAC Hybrid Materials

#### 2.3.1. Fourier Transform Infrared (FTIR) Spectra of SiO_2_/DAC

FTIR spectra of the SiO_2_/DAC hybrid materials were recorded with a FTIR spectrophotometer (Thermo Scientific Nicolet IS10, Thermo Fisher Scientific Co., Waltham, MA, USA) using potassium bromide (KBr) pellets. The measurements were performed at a data acquisition rate of 2 cm^−1^ per point and in the range from 400 to 4000 cm^−1^. To obtain a nice signal-to-noise ratio, 48 scans were performed for each sample.

#### 2.3.2. X-ray Diffraction (XRD) of SiO_2_/DAC

The X-ray diffraction patterns of the SiO_2_/DAC hybrid materials were recorded using Cu Kα radiation (λ = 0.154056 nm) on a diffractometer (Panalytical X’pert Pro MPD, PANalytical B.V. Co., Almelo, The Netherlands) at a scanning rate of 1°/min in the 2θ range from 5 to 70°.

#### 2.3.3. Thermogravimetric Analysis (TGA) of SiO_2_/DAC

Thermogravimetric analysis of the SiO_2_/DAC hybrid materials was performed with a thermal analyzer (Netzsch TG 209, NETZSCH Co., Selb, Germany). Samples (~2.0 mg) were heated from 30 to 800 °C (ramp of 10 °C/min) in a high-purity nitrogen atmosphere flowing at 80 cm^3^/min.

### 2.4. Cross-Linking of Collagen with SiO_2_/DAC

The freeze-dried collagen was dissolved in water acidified to pH 4.0 using dilute HCl to obtain a concentration of 6 mg/mL, and then the pH of the solution was adjusted to 11.0 by dropwise addition of NaOH (1.0 mol/L). DAC, SiO_2_/DAC hybrid materials and SiO_2_–NH_2_ in powder format were slowly added to the collagen solutions so that the final mass ratio of cross-linker/collagen reached 1/50 for all the samples. These solutions were gently stirred for 12 h at 20 °C, during which time all of the cross-linkers were dissolved, due to the effect of NaOH, and the resultant solutions were clear. The mixtures were further kept in the refrigerator for 24 h at 10 °C. The cross-linking reaction between cross-linkers and collagen were then terminated by adding excessive glycine, since the amino group in glycine is able to react with the active aldehyde groups in DAC or SiO_2_/DAC. After this step, these solutions were dialyzed against 0.05 mol/L acetic acid solution, and thus the residual cross-linkers were removed. Finally, these resultants were lyophilized in the freeze dryer. 

### 2.5. Characterization of Cross-Linked Collagen 

#### 2.5.1. Sodium Dodecyl Sulphate-Polyacrylamide Gel Electrophoresis (SDS-PAGE) of Collagens

Electrophoretic patterns were measured according to the method of Laemmli [26] with a slight modification, using 10% resolving gel and 4% stacking gel. Collagens were mixed with a sample buffer containing 10% β-Mercaptoethanol, to reach a final collagen concentration of 1 mg/mL, and the mixed solution was boiled for 5 min. After electrophoresis, the gel was stained for 45 min using 0.25% Coomassie brilliant blue R250 solution and destained using 7.5% acetic acid and 5% methanol. 

#### 2.5.2. FTIR of Collagens 

FTIR spectra of the collagen samples were recorded with a FTIR spectrophotometer (Thermo Scientific Nicolet IS10, Thermo Fisher Scientific Co., Waltham, MA, USA) using potassium bromide (KBr) pellets. The measurements were performed at a data acquisition rate of 2 cm^−1^ per point and in the range from 450 to 4000 cm^−1^. 48 scans were performed for each sample.

#### 2.5.3. Differential Scanning Calorimetry (DSC) of Collagens

The cross-linking effect on collagen imposed by the SiO_2_/DAC hybrid materials was evaluated by DSC (Netzsch DSC 200PC, NETZSCH Co., Selb, Germany). The freeze-dried collagens (~2 mg) were weighted accurately into aluminum pans and sealed; they were scanned over the range from 30 to 60 °C at a heating rate of 3 °C/min in a nitrogen atmosphere. Liquid nitrogen was used as a cooling medium, and empty pans were used as the reference. 

#### 2.5.4. Oscillatory Rheological Measurements of Collagens

The rheological measurements of solutions were performed on a Rheometer System (MARS III, HAAKE Co., Karlsruhe, Germany) equipped with a cone-and-plate geometry (diameter 30 mm; angle 2°). For the precise control of sample temperature, temperature was controlled to within an accuracy of ±0.10 °C. The samples for oscillatory rheological tests were measured after each equilibration time for 3 min. Rheological properties were measured by dynamic frequency sweeps at a constant strain of 5% within the linear range. Dynamic frequency sweep tests for all the samples were performed from 0.1 to 10 Hz at constant temperature of 20 °C. The storage modulus (G’), loss modulus (G”) and tan δ were recorded.

#### 2.5.5. Field Emission Scanning Electron Microscopy (FESEM) of Collagens

The morphologies of lyophilized DAC, SiO_2_–NH_2_ and the modified collagen were examined with a field-emission scanning electron microscope (Nova FEI NanoSEM 230, FEI Co., Hillsboro, OR, USA), operated at an accelerating voltage of 10 or 5 kV.

## 3. Results and Discussion

### 3.1. Preparation and Characterization of SiO_2_/DAC Hybrid Materials

**Scheme 1:**
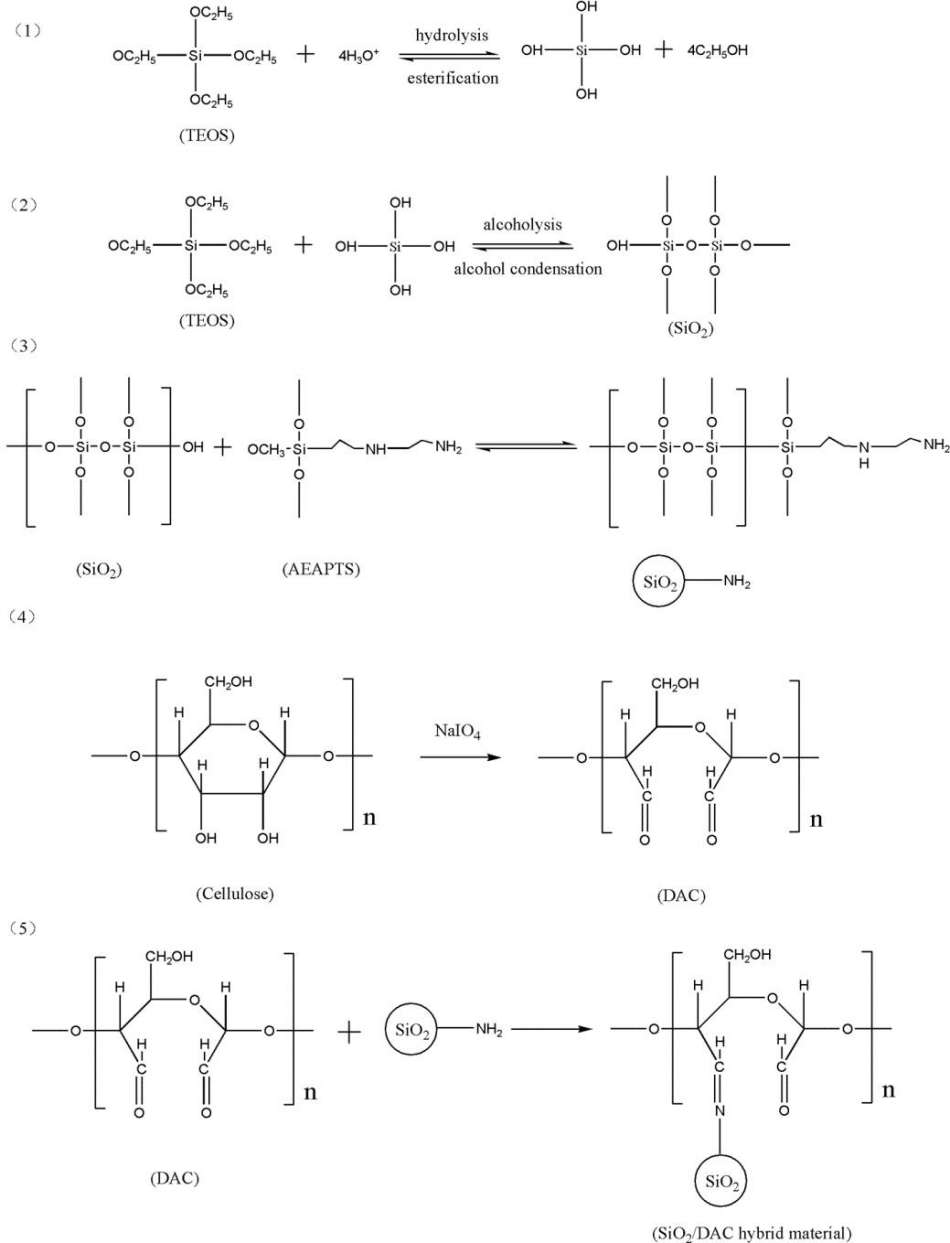
Schematic illustration of the preparation of SiO_2_/DAC hybrid materials.

Scheme 1 displays the synthetic routes of SiO_2_, DAC and the SiO_2_/DAC hybrid materials. After the Schiff base reaction between the aldehyde groups of DAC and the amino group of SiO_2_–NH_2_, SiO_2_ was immobilized onto the chains of DAC. Therefore, the prepared hybrid materials can be described as SiO_2_ immobilized DAC, possessing both of the aldehyde groups and SiO_2_ as the functional constituents reacting with collagen. 

**Figure 1:**
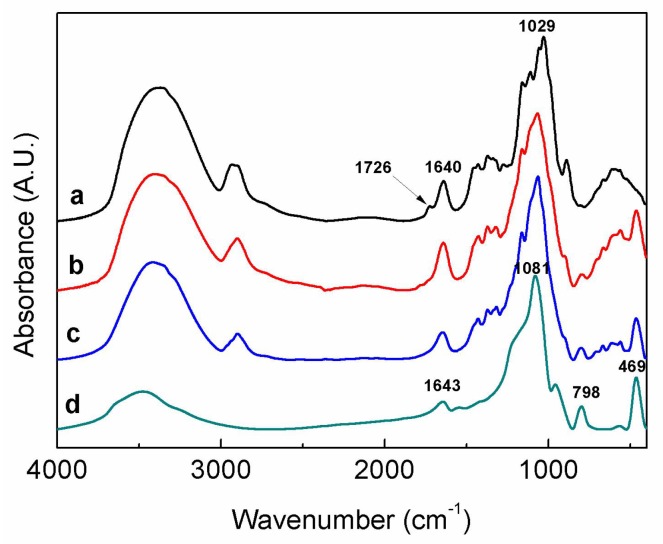
FTIR spectra of DAC (**a**), SiO_2_ (**d**) and the SiO_2_/DAC hybrid materials with ratios of 1/10 (**b**) and 1/2 (**c**).

As shown in Figure 1, the FTIR spectra of DAC showed two characteristic peaks at 1726 cm^−1^ and 880 cm^−1^, which is in line with results reported previously [27,28]. A diffuse band at 880 cm^−1^ can be assigned to the hemiacetal and hydrated form [29]. The sharp peak at 1726 cm^−1^ is a characteristic band of carbonyl groups [28]. The absorption band at 3452 cm^−1^ of the SiO_2_ indicated that there were plenty of hydroxyl groups on its surface. While the absorptions at 1081 cm^−1^, 798 cm^−1^ and 469 cm^−1^ were attributed to the antisymmetric stretching vibration, symmetric stretching vibration and flexural vibration of Si–O–Si, respectively [30]. After reaction with SiO_2_, the hybrid materials SiO_2_/DAC exhibited new characteristic peaks located at 1067, 795 and 468 cm^−1^, as well as 1060, 801 and 469 cm^−1^, for samples with SiO_2_/DAC of 1/10 and 1/2, respectively, which were derived from the absorption of Si–O–Si. It is therefore suggested that SiO_2_ was probably immobilized onto the DAC molecules.

**Figure 2:**
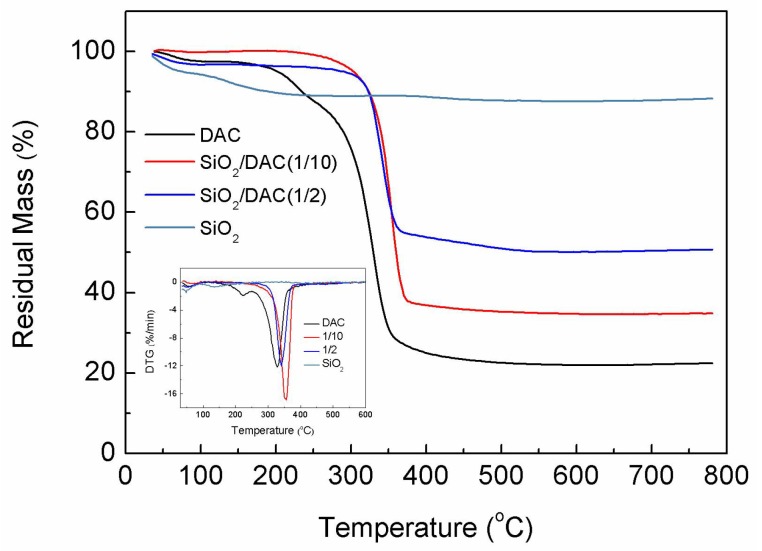
Thermogravimetric curves of the SiO_2_/DAC hybrid materials.

The TG thermographs and the DTG curves (the inserts) for samples are shown in Figure 2. As indicated by the high residual masses after the decomposition step of the TG curves, it was found that DAC had a low char yield on pyrolysis, while the char yields were increased with the increase of SiO_2_/DAC ratios. This could be attributed to the hybridization of DAC with SiO_2_–NH_2_, as the latter is an inorganic matter and exhibited a high char yield, as shown in Figure 2.

From the DTG curves, the corresponding temperature of the maximum decomposition rate (*T*_max_) could be obtained. We have previously observed that the native cellulose had a *T*_max_ of 359.6 °C [31]. After treatment with NaIO_4_, the *T*_max_ was reduced to 328.2 °C for the resultant DAC. The reduction in decomposition temperature for DAC compared to that of cellulose has been reported by Kim et al. [25]. However, the SiO_2_/DAC hybrid materials had *T*_max_ of 355.4 and 341.8 °C for SiO_2_/DAC ratios of 1/10 and 1/2, respectively, indicating that the thermal stability of DAC could be improved by the introduction of inorganic matter. It should be noted that there was no distinct weight loss peak for the pure SiO_2_, suggesting that the sample was inorganic.

**Figure 3:**
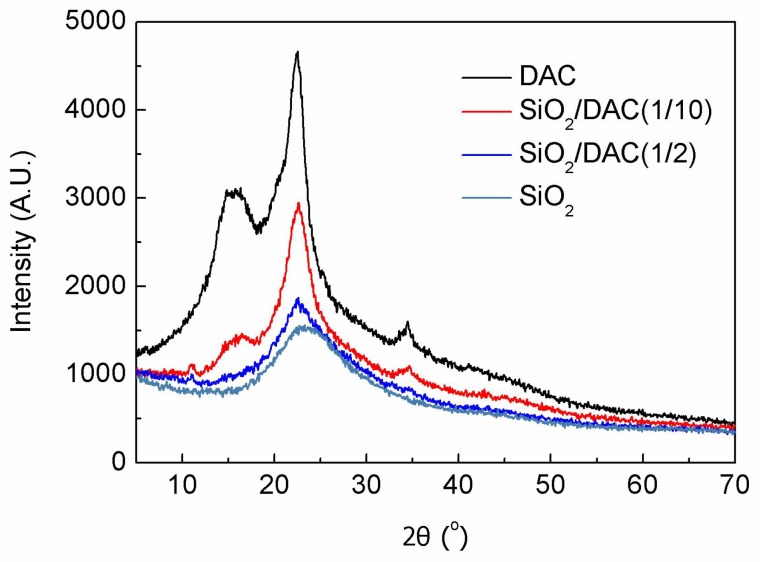
XRD patterns of the SiO_2_/DAC hybrid materials.

Figure 3 shows the XRD patterns of all the samples. For DAC, the diffraction peaks observed at 14.8°, 16.3° and 22.4° were attributed to the cellulose I pattern [32], but it was also reported that the crystallinity would be lower than that of cellulose [33]. SiO_2_ exhibited a broad peak at ~23.5°, which was in accordance with the reported result and suggested an amorphous form for SiO_2_ [34]. In the case of hybrid material with SiO_2_/DAC mass ratios of 1/10 and 1/2, the diffraction peaks disappeared significantly and disproportionately in terms of the SiO_2_/DAC mass ratios, indicating the obvious decline in the degree of crystallinity of DAC. This observation suggests that there might be an interaction between DAC and SiO_2_ particles, restricting the capacity of DAC to form a well-defined order.

### 3.2. FESEM of SiO_2_-DAC

**Figure 4:**
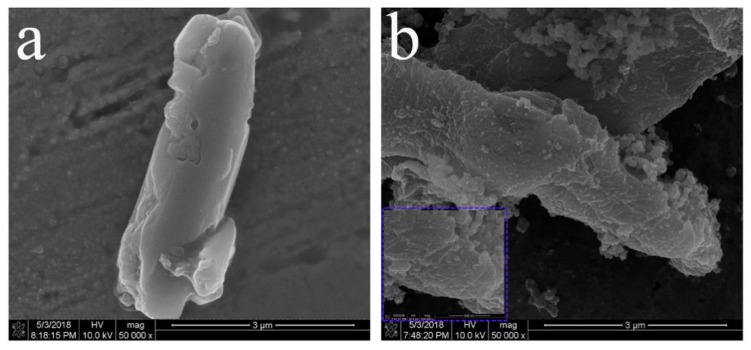
FESEM images (scale bar = 3 μm) of DAC (**a**), and SiO_2_/DAC (1/2) (**b**) (Small figure at bottom left has a magnification of 200,000 (the blue dashed outline). Bars, 500 nm.).

Figure 4 shows the FESEM images for DAC and SiO_2_/DAC (1/2). It can be seen that DAC exhibited long and short rods (Figure 4a). For SiO_2_/DAC, there were many particles of SiO_2_-NH_2_ bonded to surface of DAC, which further demonstrates that interaction occurred between the molecules of SiO_2_-NH_2_ and DAC. In addition, it was obviously found that the size of SiO_2_-NH_2_ was tens to hundreds of nanometers. For simplicity, in the following, SiO_2_ refers to SiO_2_-NH_2_.

### 3.3. The Cross-Linking of Collagen Using SiO_2_/DAC Hybrid Materials as Modifiers

**Scheme 2:**
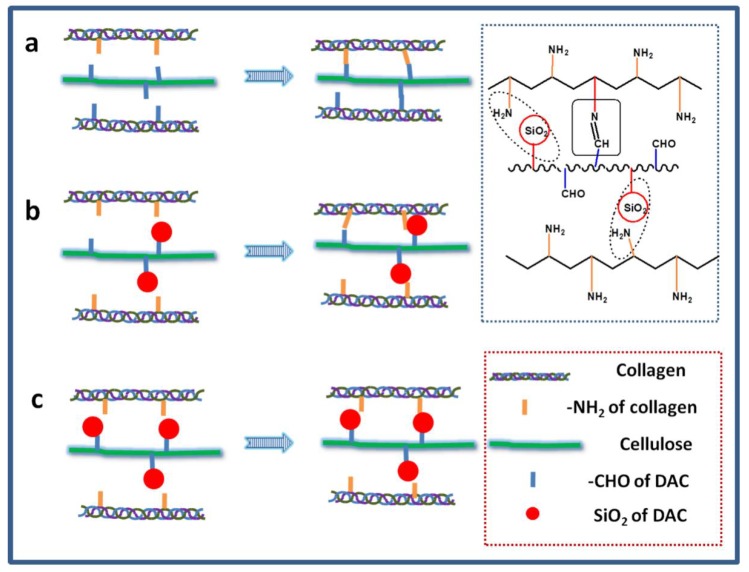
Schematic illustration of the cross-linking mechanism of collagen with SiO_2_/DAC. (**a**) Interaction between the amino groups of collagen and the paired aldehyde groups of DAC; (**b**) hydrogen bonds formed between the hydroxyl groups of SiO_2_ in SiO_2_/DAC and the hydroxyl groups or amino groups of collagen; (**c**) more SiO_2_ in SiO_2_/DAC replaced aldehyde groups and bonded to the molecules of collagen).

The prepared SiO_2_/DAC hybrid materials, as well as the pure DAC and SiO_2_, were applied in the modification of collagen solution. As the modifiers were added into collagen solution, the powders were dispersed quickly with stirring and then dissolved gradually due to the effect of basic pH. It should be pointed out that although the pH was just 11.0, DAC could be dissolved because its structure has been destroyed during the previous processes, which included hydrolysis and oxidation. After reaction, clear mixtures could be obtained. Scheme 2 presents the cross-linking mechanism of collagen with SiO_2_/DAC, which could possibly be ascribed to two types of reaction in the mixtures. Firstly, there exists the Schiff base reaction between the amino groups of collagen and the paired aldehyde groups of the cross-linking reagents [19]; secondly, the hydroxyl groups of SiO_2_ in the hybrid materials can form hydrogen bonds with the hydroxyl groups or amino groups of collagen [21]. In fact, the cross-linking mechanism might be more complex. The cross-linking products were then characterized to evaluate the influences of SiO_2_/DAC hybrid materials on the structural properties of collagen solutions.

**Figure 5:**
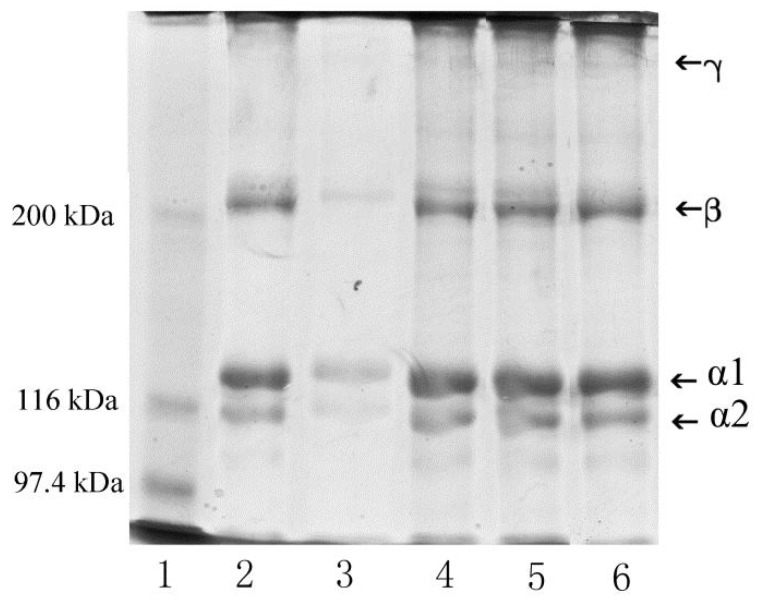
SDS-PAGE patterns of native collagen and collagens modified by the SiO_2_/DAC hybrid materials: (**lane 1**) protein markers; (**lane 2**) native collagen; (**lane 3**) collagen modified by DAC; (**lane 4**) collagen modified by SiO_2_/DAC (1/10); (**lane 5**) collagen modified by SiO_2_/DAC (1/2); (**lane 6**) collagen modified by SiO_2_.

**Figure 6:**
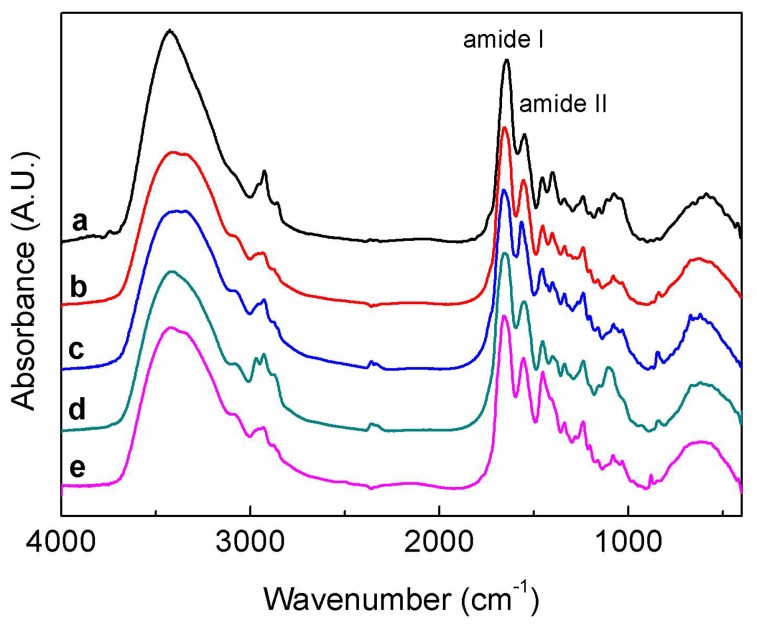
FTIR spectra of native collagen (**a**), and collagen cross-linked by the SiO_2_/DAC hybrid materials: collagen cross-linked by DAC (**b**); collagen cross-linked by SiO_2_/DAC (1/10) (**c**); collagen cross-linked by SiO_2_/DAC (1/2) (**d**); and collagen cross-linked by SiO_2_ (**e**).

SDS-PAGE was performed to identify the molecular weight distribution of collagens. As can be seen from Figure 5, the pattern for all the collagens consists of two α-chains (α1 and α2) as the major constituents. Components with higher molecular weight (MW), including β- and γ-components, were also observed. This suggests that the native triple-helix of collagen was not destroyed by the introduction of cross-links, which was confirmed by the quite similar characteristics observed between the FTIR spectra of all of the collagens (as shown in Figure 6). In the case of collagen modified by DAC, the band intensity for α-chains and β-components was significantly reduced, which could be due to the formation of cross-linked aggregates with higher MW that could not get through the gel [35]. However, as displayed in the gel patterns, the chemical cross-linking effect on collagen became weaker when SiO_2_/DAC or pure SiO_2_ were employed. 

### 3.4. DSC Thermograms of Cross-Linked Collagens

**Figure 7:**
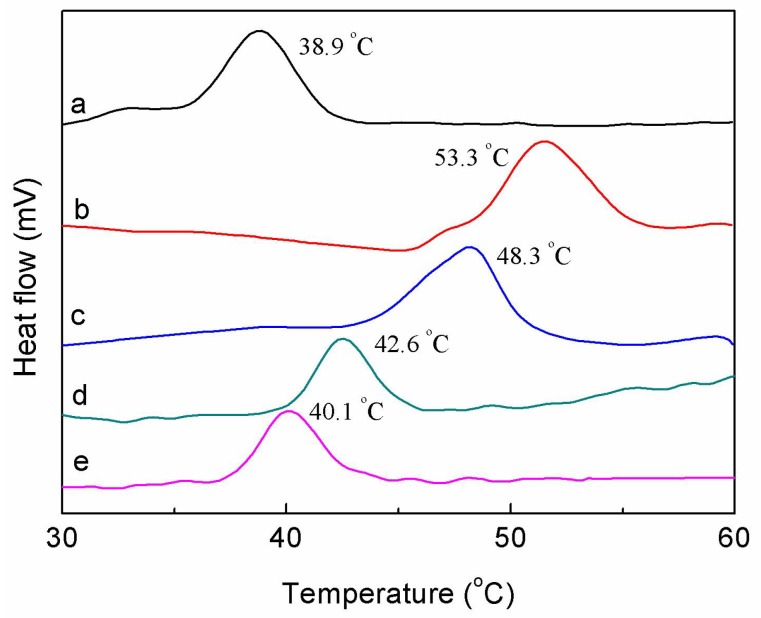
DSC thermograms of native collagen (**a**), and collagen cross-linked by the SiO_2_/DAC hybrid materials: collagen cross-linked by DAC (**b**); collagen cross-linked by SiO_2_/DAC (1/10) (**c**); collagen cross-linked by SiO_2_/DAC (1/2) (**d**); and collagen cross-linked by SiO_2_ (**e**).

DSC thermograms of native collagen and collagen modified by the SiO_2_/DAC hybrid materials are shown in Figure 7. Endothermal peaks associated with the helix-coil transition due to the thermal disruption of hydrogen bonds, which was thought to stabilize the triple helix of collagen, were detectable from the heat flow curves as a function of temperature. The un-modified collagen had a denaturation temperature (T_d_) of 38.9 °C, which was in line with previous reports [36,37]. As for collagen cross-linked by DAC, the T_d_ was significantly increased by 14.4 °C. However, the T_d_ of cross-linked collagen by SiO_2_/DAC decreased with increasing SiO_2_/DAC ratios, but was still obviously higher than that of the un-modified collagen. Note that the cross-linking of pure SiO_2_ had a slight effect on the thermal stability of collagen.

### 3.5. Dynamic Rheology Tests of Cross-Linked Collagens

**Figure 8:**
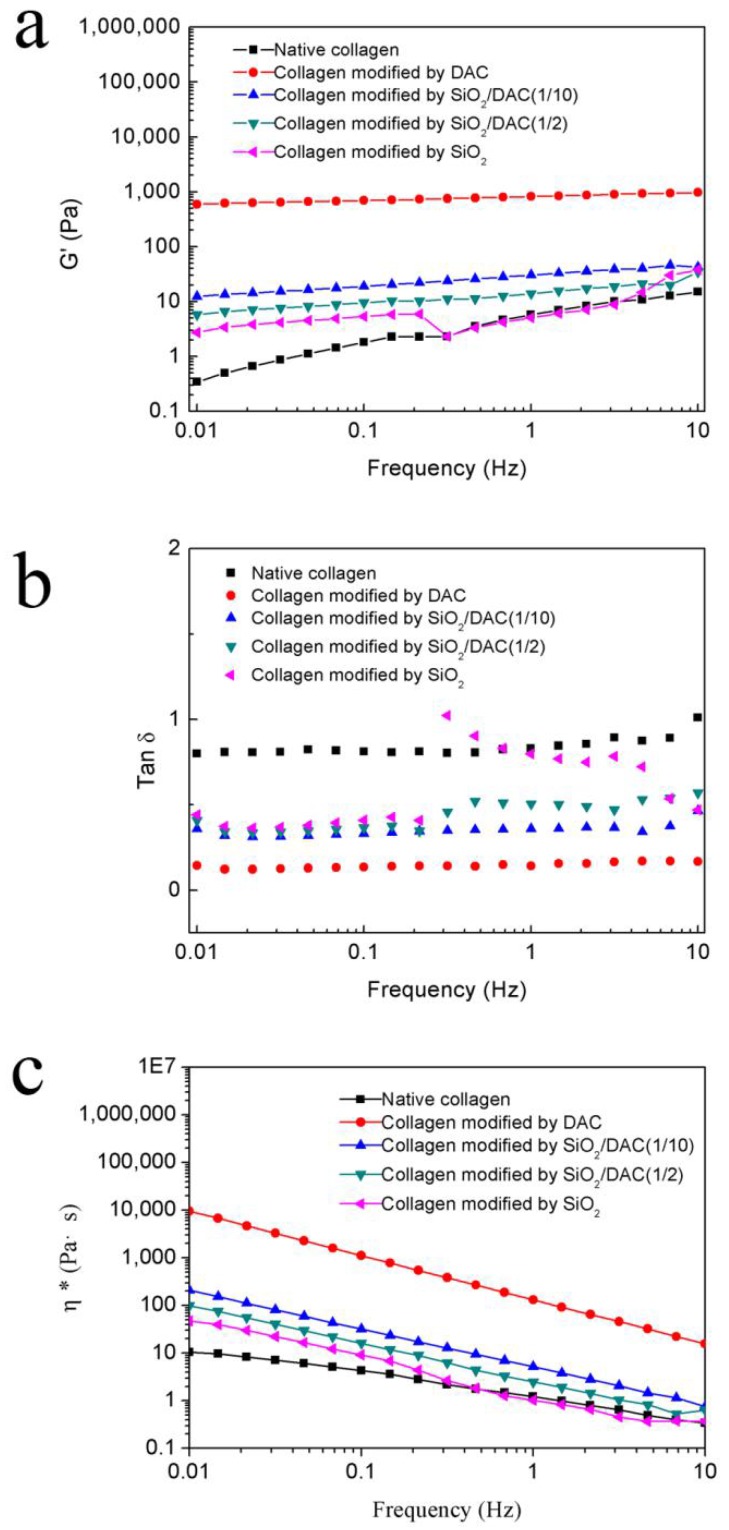
G’ (**a**), tan δ (**b**) and η* (**c**) of native collagen and collagen cross-linked by the SiO_2_/DAC hybrid materials.

It was reported that cross-linked collagen was more resistant to deformation and to flow than native collagen [38], which was reflected in its viscoelastic properties. The frequency dependence of G’, tan δ and η* for all the samples over the frequency range of 0.01–10 Hz at 25 °C is depicted in Figure 8. As can be seen from Figure 8, the values of G’ were increased exponentially with the addition of DAC. However, the values of G’ decreased remarkably for the collagen modified by SiO_2_/DAC (1/10), although they were still greater than those of the un-modified collagen. With increasing SiO_2_/DAC ratios, the values of G’ decreased continuously. It was interesting to note that collagen modified by SiO_2_ displayed a higher G’ than that of native collagen when the shearing frequency was no more than 0.2 Hz, while the values were close to those of native collagen when the shearing frequency further increased to 10 Hz. It seemed that the entanglement network of collagen was not stable enough because of the weak interactions between collagen and SiO_2_, and thus the network was easily destroyed when the samples underwent a strong mechanical treatment. 

The technique of dynamic oscillation is useful in resolving the structural properties of materials into a solid-like and a liquid-like response (G’ and G”, respectively) [39]. Generally, the ratio G”/G’, which is defined as the loss tangent (tan δ), was used to describe the flowing behavior of molecules, and it crossed the threshold (tan δ = 1) from solid-like to liquid-like behavior [40]. That is, the smaller the value of tan δ, the more rubbery or elastomeric the behavior of the material was [41]. The tan δ value of all samples was smaller than 1 under our experimental conditions, indicating a solid-like network of collagen solution. However, as DAC was added, the modified collagen exhibited tan δ values below 0.17, indicating the absolute solid-like behavior of this sample. When the SiO_2_/DAC hybrid materials were used, the tan δ of collagen increased to different extents, suggesting that the solid-like network tended to become a liquid-like network, due to the moderate cross-linking effect of the hybrid modifier. With respect to the sample modified by SiO_2_, the tanδ curves displayed a trend similar to that of G’ when the shearing frequency was increased, which again confirmed the weak interaction between collagen and SiO_2_. 

From the changes in the complex viscosity (η*) (Figure 8c), it could be seen that all samples showed a shear thinning behavior. Note that the η* values of collagen cross-linked by DAC were far larger than those for the other samples, indicating that a rapid cross-linking reaction had occurred between collagen and DAC, while the η* values of collagen cross-linked by SiO_2_/DAC or SiO_2_ decreased as the ratio of SiO_2_ in SiO_2_/DAC increased. The decrease in viscosity due to the introduction of SiO_2_ was in favor of processing for collagen solution.

### 3.6. FESEM of Cross-Linked Collagens

**Figure 9:**
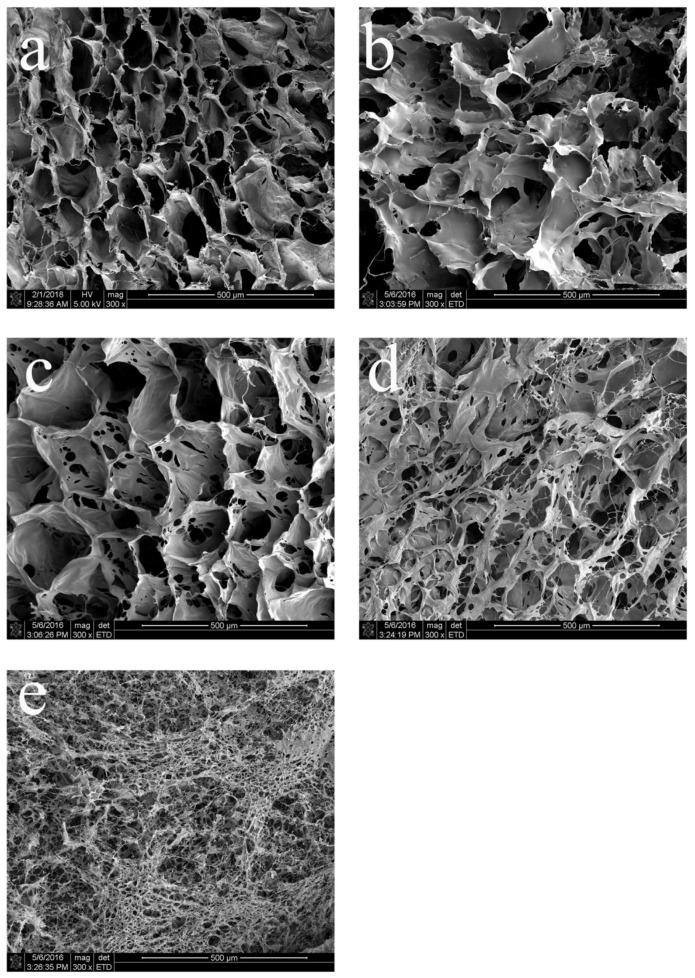
FESEM images (scale bar = 500 μm) of native collagen (**a**), and collagen cross-linked by the SiO_2_/DAC hybrid materials: collagen cross-linked by DAC (**b**); collagen cross-linked by SiO_2_/DAC(1/10) (**c**); collagen cross-linked by SiO_2_/DAC(1/2) (**d**); and collagen cross-linked by SiO_2_ (**e**).

The FESEM images, as shown in Figure 9, displayed a well-ordered porous structure for native collagen (Figure 9a). As for collagen modified by DAC (Figure 9b), the sponge had a heterogeneous structure, which was a result of the excessively fast cross-linking effect. It could be observed from Figure 8b that the permeability of pores was decreased, and the aggregation of collagen was enhanced due to the cross-linking effect of DAC. In contrast, with the increased ratio of SiO_2_/DAC, the morphological characteristics of porosity and fibrillar network of modified collagen became evident, particularly for the sample modified by SiO_2_/DAC(1/2) (Figure 9d), suggesting that a moderate cross-linking effect on collagen could be imposed by the hybrid modifier. It should be noted that collagen modified by the pure SiO_2_ had a distinct morphology compared to the other samples; that is, a large number of fibers were visible. This morphology was quite similar to that reported previously [34], indicating that the aggregation behavior of collagen was influenced by the addition of nano-SiO_2_.

## 4. Conclusions

A novel cross-linking agent of collagen was prepared via the hybridization of DAC and nano-SiO_2_. The structure of the SiO_2_/DAC hybrid materials was studied. Then, SiO_2_/DAC hybrid materials were added into collagen solutions. Multiple sources of evidence were provided that the triple-helix of collagen was not destroyed; rather, the thermal stability could be significantly improved due to the interactions between SiO_2_/DAC and collagen. Compared to collagen modified by pure DAC, collagen modified by the SiO_2_/DAC hybrid materials exhibited a better flowability, protecting collagen solutions against excessive gelation. This will be of significance for the development of new cross-linkers and for the process and design of materials based on collagen in the format of solution.
